# Direct TEM observations of growth mechanisms of two-dimensional MoS_2_ flakes

**DOI:** 10.1038/ncomms12206

**Published:** 2016-07-14

**Authors:** Linfeng Fei, Shuijin Lei, Wei-Bing Zhang, Wei Lu, Ziyuan Lin, Chi Hang Lam, Yang Chai, Yu Wang

**Affiliations:** 1 Department of Applied Physics, The Hong Kong Polytechnic University, Hong Kong, China; 2 School of Materials Science and Engineering, Nanchang University, Nanchang, Jiangxi 330031, China; 3 School of Physics and Electronic Sciences, Changsha University of Science and Technology, Changsha 410004, China

## Abstract

A microscopic understanding of the growth mechanism of two-dimensional materials is of particular importance for controllable synthesis of functional nanostructures. Because of the lack of direct and insightful observations, how to control the orientation and the size of two-dimensional material grains is still under debate. Here we discern distinct formation stages for MoS_2_ flakes from the thermolysis of ammonium thiomolybdates using *in situ* transmission electron microscopy. In the initial stage (400 °C), vertically aligned MoS_2_ structures grow in a layer-by-layer mode. With the increasing temperature of up to 780 °C, the orientation of MoS_2_ structures becomes horizontal. When the growth temperature reaches 850 °C, the crystalline size of MoS_2_ increases by merging adjacent flakes. Our study shows direct observations of MoS_2_ growth as the temperature evolves, and sheds light on the controllable orientation and grain size of two-dimensional materials.

## Abstract (web summary)

MoS_2_ nanostructures are potentially useful in electronic applications. Here, Fei *et al.* show using *in situ* electron microscopy that MoS_2_ grows in a vertical manner at low temperature, then rotates to horizontal structures, and finally develops in size by merging adjacent flakes at high temperature.

## Graphical abstract

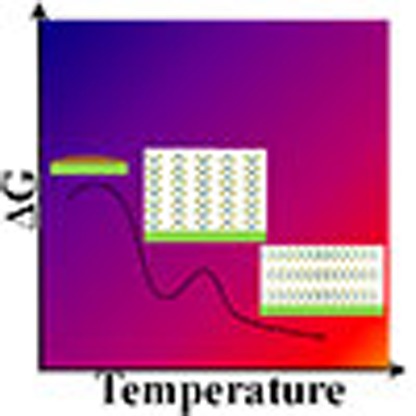

Two-dimensional (2D)-layered MoS_2_ has shown great potential for various applications, including electronics^1^,^2^,^3^,^4^,^5^, optoelectronics^6^,^7^,^8^,^9^, photonics^10^,^11^,^12^, sensors^13^,^14^,^15^,^16^, catalysis^17^,^18^,^19^,^20^,^21^, biomedicine^22^ and energy storage^23^,^24^,^25^. Interestingly, the terrace and the edge sites of MoS_2_ exhibit specific physical and chemical properties^26^,^27^. Horizontal MoS_2_ flakes with the basal plane exposed, possessing lots of terrace sites and few dangling bonds, have been demonstrated for field-effect transistors and photoelectric devices^4^,^7^. In contrast, vertically aligned MoS_2_ structures with high-density edge sites, which are abundant with *d*-orbital electrons that facilitate the bonding with other elements, have been used for hydrogen evolution reaction, oxygen evolution reaction and gas adsorption^20^,^21^. It is a crucial issue to control the orientation of MoS_2_ in the horizontal or vertical direction on substrates for different applications. Chemical vapour deposition (CVD) is regarded as a deterministic method for controllable fabrication of 2D materials^28^,^29^,^30^,^31^ by which both the vertical and the horizontal MoS_2_ structures have been fabricated^32^,^33^, and there was also effort in revealing the underlying growth mechanism of MoS_2_ (ref. 34). Compared with sulfurization of Mo, MoO_3_ and MoCl_5_, the growth of MoS_2_ by the thermolysis of ammonium thiomolybdates ((NH_4_)_2_MoS_4_) has the advantages of single-precursor source, large growth window and the potential for mass production^35^. However, it still remains unclear how to control the orientation (vertical or horizontal) and grain size of MoS_2_ flakes by the thermolysis of (NH_4_)_2_MoS_4_.

Technical advances in transmission electron microscopy (TEM) with high spatial and temporal resolution allow us to monitor the real-time growth processes at an atomic scale and to understand the growth mechanism of low-dimensional materials^36^,^37^. Significant progresses have been made in figuring out the mechanisms behind sophisticated physicochemical processes using dedicated TEMs, such as the CaCO_3_ nucleation^38^, Pt_3_Fe nanoparticles' attachment^39^ and Y_2_BaCuO_5_ nanowire growth^40^. TEM observations of the growth of 2D materials remains rather limited because of the critical challenges on obtaining high-resolution images at high temperature while maintaining the 2D materials' growth in steps.

In this work, we use a heating stage with a Si_3_N_4_ membrane inside TEM to *in situ* observe the thermolysis of (NH_4_)_2_MoS_4_ and the subsequent crystallization behaviour of MoS_2_. We show that vertically aligned MoS_2_ flakes can be grown by a rapid temperature ramp, and further transit into horizontally aligned MoS_2_ flakes with the increasing temperature, which can be understood as a result of the minimization of the system energy, in good agreement with our theoretical calculations. The grain size of MoS_2_ flakes can be enlarged through oriented attachment and Ostwald ripening mechanisms by further increasing growth temperature or providing more precursors.

## Results

### Selected-area electron diffraction evolved with temperature

**Figure 1:**
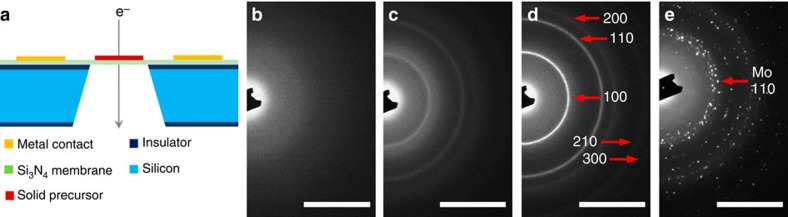
SAED patterns evolved with growth temperature. (**a**) Schematic side view of the experimental set-up of *in situ* TEM heating stage. (**b**–**e**) The evolution of SAED patterns across a wide temperature range: (**b**) 25 °C, (**c**) 400 °C, (**d**) 780 °C and (**e**) 900 °C. Scale bars, 5 nm^−1^.

To dynamically monitor the thermolysis of (NH_4_)_2_MoS_4_ and the crystallization process of MoS_2_ inside TEM (see Fig. 1a and Supplementary Fig. 1, the experimental design for this work), we first carried out electron diffraction analysis across a wide temperature range, from room temperature to 900 °C. Figure 1b–e shows the evolution of selected-area electron diffraction (SAED) patterns with the increasing temperature. From room temperature to 400 °C, no diffraction pattern is exhibited (Fig. 1b), indicating that the precursor is kept at its amorphous state. When the temperature reaches 400 °C, blurred diffraction rings emerge (Fig. 1c). The SAED patterns in Fig. 1c can be well indexed to a MoS_2_ structure with the space group of P63/mmc (JCPDS card No. 77-1716), implying that crystallization is initialized from this stage. Supplementary Fig. 2 shows the energy-dispersive X-ray spectra (EDS) acquired at room temperature and 400 °C. The increased atomic ratio of Mo/S from 1/4 (at room temperature) to 1/2 (at 400 °C) further confirms the decomposition of (NH_4_)_2_MoS_4_ into MoS_2_ at 400 °C. It has been reported that the thermolysis of (NH_4_)_2_MoS_4_ in an N_2_ environment resulted in the production of MoS_2_ via the following equations^35^,^41^:


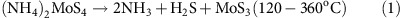



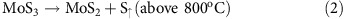


The simultaneous thermogravimetric and differential scanning calorimetry (TGA/DSC) analysis of (NH_4_)_2_MoS_4_ precursor under flowing N_2_ (Supplementary Fig. 3) also verifies the foregoing chemical reactions. The different MoS_2_ formation temperatures in TEM and TGA/DSC (400 °C in TEM, while above 800 °C in TGA/DSC) can be ascribed to the different heating atmospheres. The temperature for forming MoS_2_ in high vacuum (TEM chamber) is relatively lower than that in a protective gas flow (in TGA/DSC).

With the increasing temperature, the diffraction rings became sharper, brighter and more discrete (also refer to Supplementary Fig. 4), suggesting the improvement of crystallinity and the increase in the crystal size. While the temperature reaches up to 900 °C, the continuous diffraction ring becomes blurred and a lot of bright spots appear (Fig. 1e). The emerging diffraction spots belong to metallic Mo (JCPDS card No. 89-5156), which is very likely to be resulted from the decomposition of MoS_2_ at high temperature (MoS_2_→Mo+2S_↑_). On the basis of these observations, we can propose that the crystallization of MoS_2_ occurs at 400–900 °C in vacuum, and the MoS_2_ crystals grow rapidly in size after 800 °C. The subsequent analysis is to sequentially capture systematical high-resolution TEM (HRTEM) micrographs as a function of increasing temperatures in this range.

### Layer-by-layer growth of vertical MoS_2_ structures

**Figure 2:**
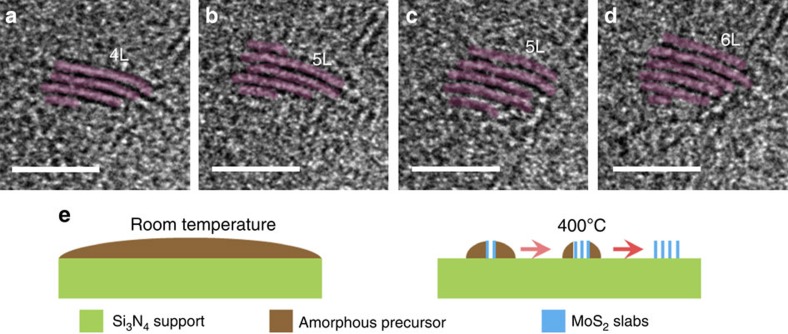
Layer-by-layer growth dynamics of the vertical MoS_2_ structure at 400 °C. (**a**–**d**) Time-resolved *in situ* TEM micrograph series at (**a**) 0 s, (**b**) 12 s, (**c**) 37 s and (**d**) 51 s. Frames captured from Supplementary Movie 1. 4L, 5L and 6L stand for four-layer-, five-layer- and six-layer MoS_2_ structures, respectively. Coloured sketches are included to illustrate the MoS_2_ layers. Time listed for all figures is relative to the beginning of Supplementary Movie 1. Scale bars, 5 nm. (**e**) Schematic illustrations of the growth process at room temperature (left panel) and 400 °C (right panel).

**Figure 3:**
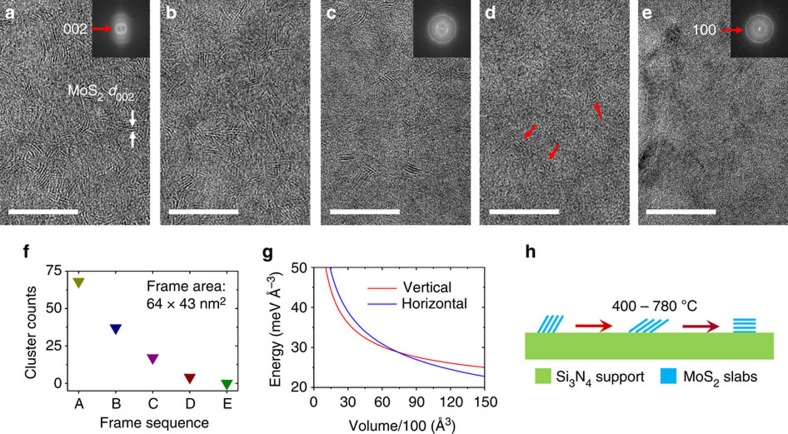
Vertical-to-horizontal transition from 400 to 780 °C. (**a**–**e**) TEM images versus the increasing temperatures: (**a**) 400 °C, (**b**) 500 °C, (**c**) 600 °C, (**d**) 700 °C and (**e**) 780 °C. The insets in **a**,**c**,**e** are the corresponding fast Fourier transform (FFT) patterns. The red arrows in **d** denote the vertical MoS_2_ structures at 700 °C to provide better illustration. Scale bars, 20 nm. (**f**) Statistical distribution of the counts of the vertical MoS_2_ flakes in frames **a**–**e**. (**g**) The energy density of MoS_2_ structures along two orientations as a function of their volumes, showing the competition of total system energy between the vertical and horizontal structures during crystal growth. (**h**) Schematic process of the vertical-to-horizontal transition.

At 400 °C, the sample is found to be slightly crystallized into loosely packed small clusters (1–3 nm) from the original amorphous precursor. Figures 2 and 3a show that the (002) edge sites of MoS_2_ are exposed, implying that the MoS_2_ slabs adopt a vertical growth and stand upright on the Si_3_N_4_ support at this stage. The interlayer distance of MoS_2_ slabs is ∼0.6 nm, similar to the value in bulk counterpart. Although the surface energy of the MoS_2_ edge sites is larger than that of the terrace sites by 2 orders of magnitude^42^, our result is in accordance with the previous *ex situ* rapid growth of MoS_2_ by the sulfurization of Mo layers, in which the MoS_2_ structure also adopts a vertical orientation in certain circumstances^32^,^33^.

We further captured a movie (Supplementary Movie 1) to vividly describe the dynamics of the vertical growth of MoS_2_ layers at 400 °C, and the snapshot frames are presented as Fig. 2a–d, exhibiting a layer-by-layer vertical growth. Figure 2a shows a four-layered vertical MoS_2_ slab surrounded by the amorphous precursor. With the continuous heating, the monomers are attached on the existing MoS_2_ cluster, and subsequently grow new slab to match the size of the existing ones. It is noteworthy that the growth of new layers is initialized from the step edges of the previous ones because step edges are more energetically active (see Fig. 2b,d). This finding is partially matched to the earlier observations by Helveg *et al.*^34^, in which new MoS_2_ slab started growth on old ones from either the middle part or the edge part. In addition, our results also suggest that the MoS_2_ cluster shows negligible in-plane growth throughout our observations, as shown in Fig. 2a–d, because the formation of new layers is more energetically favourable than the elongation of the existing layers. The whole process is schematically illustrated in Fig. 2e.

### Vertical-to-horizontal transition

As shown in Fig. 3a–e, it is remarkable that the exposed MoS_2_ (002) planes gradually disappear instead of getting larger as the growth temperature continuously increases. The insets in Fig. 3a,c,e present the corresponding fast Fourier transform patterns, showing a continuous decrease in the brightness of the (002) halo ring; in contrast, the brightness of the (100) halo ring greatly increases (also see Fig. 3f for the statistical counts of vertical MoS_2_ structures throughout Fig. 3a–e). This implies that the preferred orientation of MoS_2_ on Si_3_N_4_ is changed from the vertical direction towards the horizontal one, namely, exposing their terrace sites instead of edge sites. The orientation of the MoS_2_ slabs with respect to the electron beam has a determinative impact on the TEM contrast. The substantial image contrast of MoS_2_ (002) basal plane can only be retained while it is ±9° within the electron beam direction; larger tilted angle weakens the contrast and the slabs become unrecognizable from the support substrates^34^,^43^,^44^. Therefore, it is reasonable to infer that a grain rotation process happens at this stage (see also Supplementary Fig. 5). The MoS_2_ clusters rotated from vertical to horizontal on the substrate during the heating between 400 and 780 °C, as depicted in Fig. 3h. We repeatedly grew MoS_2_ at 780 °C and found that the as-grown polycrystalline MoS_2_ flakes are horizontally orientated, in good agreement with previous CVD growth results from either gas-phase or solid-phase precursors at similar temperatures^30^,^45^.

The initial formation of vertically aligned MoS_2_ structures and its subsequent rotation to horizontal structure are very likely to be driven by reducing total system energy and eliminating crystal defects. To investigate the evolution of total system energy during the transition, we performed the first-principles calculations concerning the surface energy and the interfacial energy between MoS_2_ and Si_3_N_4_ substrates, which are two major factors contributing to the total system energy. First, the surface energy during the transition from vertical structure (corresponding to the surface with the Miller index (100)) to horizontal structure (corresponding to the (001) surface is calculated). Our calculation results show that the (001) surface has much lower surface energy (0.0277, eV Å^−2^) than (100) surface in the rational chemical potential region. The sulfur-terminated structure (that is, (100)-S2) with the surface energy of 0.1794, eV Å^−2^ is found to be the most stable termination in the surfaces with the (100) Miller index. Second, the interfacial energy between MoS_2_ and Si_3_N_4_ substrates are different for vertical and horizontal MoS_2_ structures because the dangling bonds are present in the (100) surface but absent in the (001) surface. The interfacial energies for vertical and horizontal structures are −0.1173 and −0.0046, eV Å^−2^, respectively. Clearly, the interfacial energy for vertical MoS_2_ structure is much smaller than the horizontal structure.

Generally, the interfacial energy reduces the system energy, while the surface energy raises it. As shown above, the surface energy of vertical MoS_2_ structure is larger than that of horizontal structure; however, the interfacial energy is much smaller. Therefore, the competition between surface energy and interfacial energy of MoS_2_ structures determines the preferred growth orientation. To illustrate this mechanism, we evaluate the energies of MoS_2_ structures in different orientations as a function of the volumes. Since the height of the structure is limited in the growth of vertical MoS_2_, the energy is minimized with a constraint, in which the height of particle is less than 15 Å. The corresponding energy density, the optimal length, width and height of different surface orientations as a function of volumes are summarized in Fig. 3g and Supplementary Fig. 6 (see also Supplementary Note 1). We can find that the vertically aligned MoS_2_ prefers to adopt a box with large length and height but small width, whereas the horizontally aligned MoS_2_ tends to lateral growth, in which the length, which is equal to width, is much larger than height. As shown in Fig. 3g, at the beginning of crystallization, the size is small, and hence the vertical structure has lower energy than the horizontal one. The energy difference increases with volumes and the height of vertical structure also increases. Since the height of structure is limited in the subsequent growth, the energy difference between the two orientations becomes small. When the volume is larger than the critical value, the horizontal structure becomes more stable than the vertical growth. This simplified thermodynamic model on the basis of surface energy and interfacial energy from density functional theory calculation is well consistent with the above experimental results, implying the feasibility of a vertical-to-horizontal transition during the growth (Fig. 3h).

Furthermore, defects may also play an important role in the aforementioned vertical-to-horizontal transition. At the initial stage (for example, Fig. 3a at 400 °C), the twisty MoS_2_ fringes in the HRTEM image imply substantial presence of in-plane defects (for example, dislocations, localized disordering and amorphization), resulting from a low-degree crystallization. These defects further increase the (001) surface energy on the basis of the above computation. Upon this circumstance, the vertical structure is more energetically stable than the horizontal one. However, accompanied by the rising temperature (that is, better crystallinity), the amount of defects will consequentially decrease (verified by that the lattice fringes are getting less twisty throughout Fig. 3a–d), leading to a synchronized decrease in (001) surface energy, and, hence, a vertical-to-horizontal transition.

### Formation of horizontal MoS_2_ flakes

**Figure 4:**
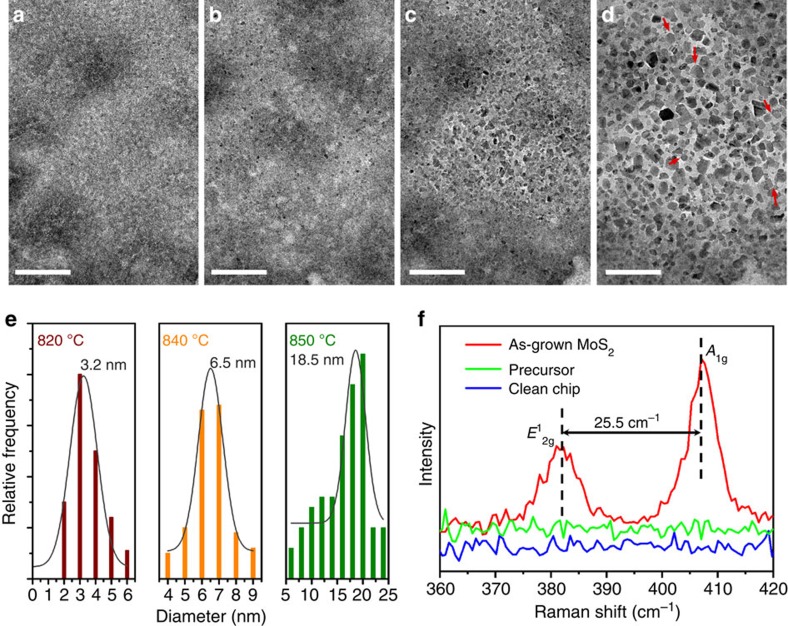
Formation of 2D MoS_2_ flakes during the heating above 800 °C. (**a**–**d**) Typical TEM image frames showing the size of MoS_2_ increases with the rising temperatures: (**a**) 800 °C, (**b**) 820 °C, (**c**) 840 °C and (**d**) 850 °C. The red arrows in **d** denote 120° angles. Scale bars, 100 nm. (**e**) Statistical distribution of the size of MoS_2_ flake as a function of the heating temperature from **b**–**d**. Frame areas, 340 × 516 nm^2^. Each histogram has been fitted with the Gaussian function. (**f**) Raman spectra of the E-chip, the (NH_4_)_2_MoS_4_ precursor and the as-grown MoS_2_.

**Figure 5:**
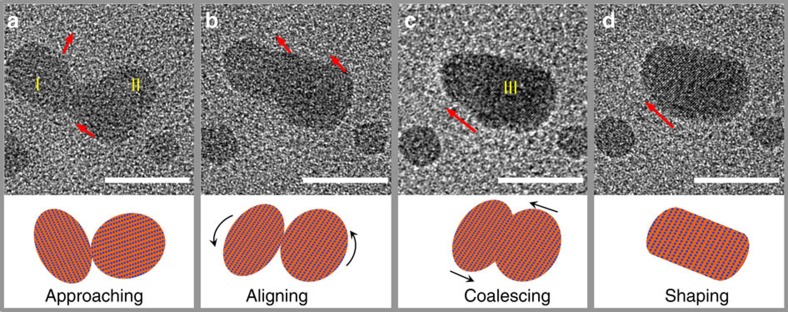
Time-resolved TEM images of the oriented attachment occurred at 840 °C. (**a**–**d**) Sequences of *in situ* TEM images indicating that flakes I and II merge into flake III; (**a**) 0 s, (**b**) 22 s, (**c**) 42 s and (**d**) 59 s. The red arrows denote the [103] direction for the adjacent flakes. Time listed for all figures is relative to the first frame. Schematic representations of the attachment stages are also included in **a**–**d**. Scale bars, 10 nm.

Thereafter, MoS_2_ particles began to precipitate from 820 °C, as shown in Fig. 4 and Supplementary Fig. 7. The red arrows in Supplementary Fig. 7A denote the precipitated MoS_2_ crystals. The average grain size of MoS_2_ crystals is enlarged with the increase in the temperature (820–850 °C, Fig. 4 and Supplementary Fig. 7), namely, 3.2 nm at 820 °C, 6.5 nm at 840 °C and 18.5 nm at 850 °C. Figure 4e shows the histograms of the MoS_2_ flake size as a function of the increasing temperature, in line with our previous SAED analysis in Fig. 1 and Supplementary Fig. 4. Moreover, the dynamics of the increased flake size was recorded and presented in Fig. 5 and Supplementary Movie 2. In Fig. 5a, two isolated MoS_2_ flakes (I and II), with exposed (103) planes but different orientations, are in contact with each other. These two flakes experienced obvious rotation of crystal orientation to match and align their (103) planes (Fig. 5b) before the disappearance of their grain interface (Fig. 5c). Finally, the coalescence of flakes I and II produces a new flake (III) with relaxed surface (Fig. 5d). The foregoing process refers to typical oriented attachment mechanism^37^,^46^,^47^. This mechanism differs from conventional crystal growth mechanism, in which high-energy facets grow faster via monomer-by-monomer attachment. We also notice that the conventional Ostwald ripening growth pathway also coexists in the process by directly providing monomers, as shown in Supplementary Movie 2, where the small particle fed the large one and became even larger^48^. Finally, when the temperature is raised to 850 °C, the shape of the MoS_2_ crystals experienced a dramatic change by facet development via mass redistribution. A majority of the MoS_2_ crystals reshaped to closely packed, quasi-hexagonal nanoflakes with faceted outlines (see Fig. 4d and Supplementary Fig. 7C, the red arrows in Fig. 4d denoted 120° angles), in good consistence with the CVD result using (NH_4_)_2_MoS_4_ in dimethylformamide as the gas-phase precursor^49^. The atomic-resolution HRTEM image in Supplementary Fig. 7c corresponds to highly crystalline nature of these nanoflakes, and the associated fast Fourier transform pattern (inset in Supplementary Fig. 7C) displayed a typical sixfold symmetry expected for MoS_2_ along its [001] direction. Figure 4f shows the *ex situ* Raman spectra of the fresh E-chip, the (NH_4_)_2_MoS_4_ precursor and the as-grown MoS_2_ (annealed at 850 °C for 30 min). No Raman signal is observed from the E-chip or the precursor, while two characteristic Raman modes of MoS_2_, the in-plane mode *E*^1^_2g_ and out-of-plane mode *A*_1g_, are clearly exhibited in the spectrum of the as-grown sample. Notice that the peak intensity of *E*^1^_2g_ is over 50% of the *A*_2g_ peak, indicating the horizontal growth of the MoS_2_ structure^32^,^50^. The separation of the two Raman peaks is ∼25.5 cm^−1^, suggesting that the MoS_2_ are four to five layers^51^, in good agreement with our *in situ* observations.

## Discussion

The lateral size of the MoS_2_ nanoflakes in our experiment is only around tens of nanometres, which is close to that by previous CVD growth and hinders its applications for transistors, sensors and so on. This small size is possibly resulted from the limited source and the restricted mass transport ability of MoS_2_ species in the all-solid environment on the substrate surface^35^. To evaluate the possibility of growing relative large-size flakes using this method, we further carried out a secondary *in situ* growth on the same E-chip, and the results are summarized as Supplementary Fig. 8. Evidently, the second growth of MoS_2_ follows the same mechanism as revealed above (refer to discussions of Supplementary Note 2). The resulting flakes are much larger than the first growth in size (from an average size of ∼20 nm after the first growth to ∼35 nm after the second growth, corresponding to an increase of ∼75%). This implies the potential in growing large-size flakes under the condition that sufficient sources are provided. In this perspective, the as-revealed mechanism should be of general applicability in other synthesis approaches (for example, CVD method) leading to MoS_2_ microsized flakes or even other 2D-layered transition metal dichalcogenides.

Hence, the overall growth mechanism for MoS_2_ from solid precursor at high temperature was disclosed. The growth can be divided into two stages: first, the low-temperature vertical stage (below 800 °C, Fig. 3, and the schemes in Figs 2, 3) and, second, the high-temperature horizontal stage (above 800 °C, Fig. 4). At the low-temperature region, the MoS_2_ growth generally includes the decomposition of solid precursor, the layer-by-layer growth of vertically aligned structures and the successive vertical-to-horizontal transition. Afterwards, at the high-temperature region, the MoS_2_ nanocrystals spontaneously precipitate, increase the crystalline size via multiple growth pathways and form hexagonal nanoflakes.

In summary, we observed the dynamic growth of 2D MoS_2_ structure on an amorphous substrate by using an *in situ* TEM upon heating a solid precursor. We microscopically identified a two-step mechanism during the crystallization of MoS_2_. Our study using *in situ* TEM technique provides fundamental understanding on synthesis of the emerging 2D materials and paves the way to rational design of functional nanostructures.

## Methods

### Sample preparation

High purity of (NH_4_)_2_MoS_4_ (Sigma-Aldrich, 99.97%) was dissolved in dimethylformamide to form a 1 wt% solution, which was then sonicated for 10 min before being used.

### TEM observation

The *in situ* growth experiment described in this work was conducted on a Protochips Aduro double-tilted platform using heating E-chip specimen support that provides atomic resolution at a thermal ramping rate of up to 10^6 ^°C s^−1^ with highly accurate temperature control of specimen inside a TEM. The TEM sample was prepared by drop-casting the above-mentioned solution on a Si_3_N_4_ membrane (Si_3_N_4_ thickness of ∼50 nm) supported by a silicon E-Chip, which was then naturally dried in air. TEM observations were conducted on a JEM-2100F field-emission transmission electron microscope operating at 200 kV, equipped with an Oxford INCA x-sight EDS Si(Li) detector. Before every experiment, the specimen was heated to 100 °C using 10 °C s^−1^ increments, and stayed at this temperature for 15 min to remove any possible organic residuals. For the initial SAED survey, the sample was heated from 100 to 900 °C at a rate of 1 °C s ^1^ and 5 min holding time at every 10 °C to identify the important temperature points. For subsequent TEM/HRTEM analysis, the sample was heated from 100 to 850 °C at a rate of 1 °C s^−1^ and 30 min holding time at each temperature for a detailed observation. Such *in situ* experiments were implemented twice to confirm the structural evolution of MoS_2_. During the observations, the sample was irradiated with a focused electron beam with a current density of *ca.* 65 pA cm^−2^ (measured from the fluorescent screen), and the image was recorded with a Gatan SC1000 ORIUS CCD camera with a short exposure time (∼1 s). The electron beam was blanked whenever possible to minimize beam effects on the sample. All reported temperatures are based on the Protochips calibration. It is also essential to assess that the *in situ* growth is a result of thermally assisted evolution or electron-induced process. Therefore, we designed control experiments with and without constant electron beam irradiation on the samples, and systematically investigated the effects of the electron beam irradiation on the growth dynamics. The detailed procedure and results are described in Supplementary Fig. 9 and Supplementary Note 3.

### Other characterizations

Simultaneous TGA/DSC analysis were performed on a NETZSCH STA 449 C Jupiter System under flowing N_2_. Raman measurement was taken using a Horiba Jobin Yvon LabRAM HR System with a laser wavelength of 488 nm. The laser spot size is ∼1 μm with 1.48 mW power. The Raman spectrum was collected with a × 100 Olympus objective lens with a numerical aperture of 0.8, and the acquisition time was set to 5 s. The matched CCD (charge-coupled device) is CCD-7041 from Horiba Jobin Yvon. The Si peak at 520 cm^−1^ was used for precalibration in the experiments.

### Theoretical calculations

The density functional theory calculations have been performed by using the Vienna *ab initio* simulation package code^52^,^53^ within the projector augmented-wave method^54^,^55^. General gradient approximations in the Perdew–Burke–Ernzerhof implementation^56^ were chosen for the exchange correlation function. A plane-wave basis set expanded in energy with a cutoff of 400 eV is used in the calculation. The surfaces have been modelled by a symmetric slab containing eight Mo atom layers and a large vacuum of at least 15 Å. The corresponding oxygen layers are used in the calculation according to different termination and surface orientations. To compare the stability of surfaces with different Miller indices, we calculated their surface free energy following the approach developed by Reuter and Scheffler^57^. Taking hexagonal Si_3_N_4_ (space group P63) as an illustrative substrate, we also calculated the interfacial energy of both surfaces. The (2 × 1) MoS_2_ (100)/Si_3_N_4_ (001) and (2 × 2) MoS_2_ (001)/Si_3_N_4_ (001) structures are used to model the interfaces. In addition, the corresponding interfacial energies are defined by 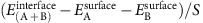, in which *E* and *S* represent the total energy and area of different systems, respectively. To determine the relative stability of the particles with different orientations, the energy of MoS_2_ particles relative to bulk MoS_2_ is also evaluated. The (100) ((001)) and (010) index planes are supposed to expose at the side of the particle with (001) ((100)) orientation. The relative energy density per volume thus can be calculated by


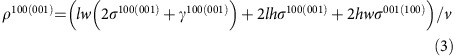


in which *l*, *w*, *h* and *v* are the length, width, height and volume of the particles, *σ*^100(001)^ and *γ*^100(001)^ represent surface energy and interfacial energy, respectively. Note that the miller index (100) ((001)) of the MoS_2_ particle used here corresponds to the vertical (horizontal) growth in article.

### Data Availability

The data that support the findings of this study are available from the corresponding author upon request.

## Additional information

**How to cite this article**: Fei, L. *et al.* Direct TEM observations on growth mechanisms of two-dimensional MoS_2_ flakes. *Nat. Commun.* 7:12206 doi: 10.1038/ncomms12206 (2016).

## Supplementary Material

Supplementary Information Supplementary Figures 1-9, Supplementary Notes 1-3 and Supplementary References

Supplementary Movie 1 Typical growth dynamics of MoS2 layer formation maintaining at 400 oC. The snapshot frames were captured as Figure 2 A-D. The video was edited to play at 4X speed.

Supplementary Movie 2 Typical growth dynamics on the coalescence of a small MoS2 nanoparticles by larger one upon heating to 840 oC. This video was edited to play at 8X speed.
