# Supplementary material for: Direct TEM observations of growth mechanisms of two-dimensional MoS2 flakes
Source: Nat Commun. 2016 Jul 14;7:12206. doi: 10.1038/ncomms12206 (PMC4947173; doi:10.1038/ncomms12206)
Supplement: Supplementary Information — Supplementary Figures 1-9, Supplementary Notes 1-3 and Supplementary References [file ncomms12206-s1.pdf]

Supplementary Figures

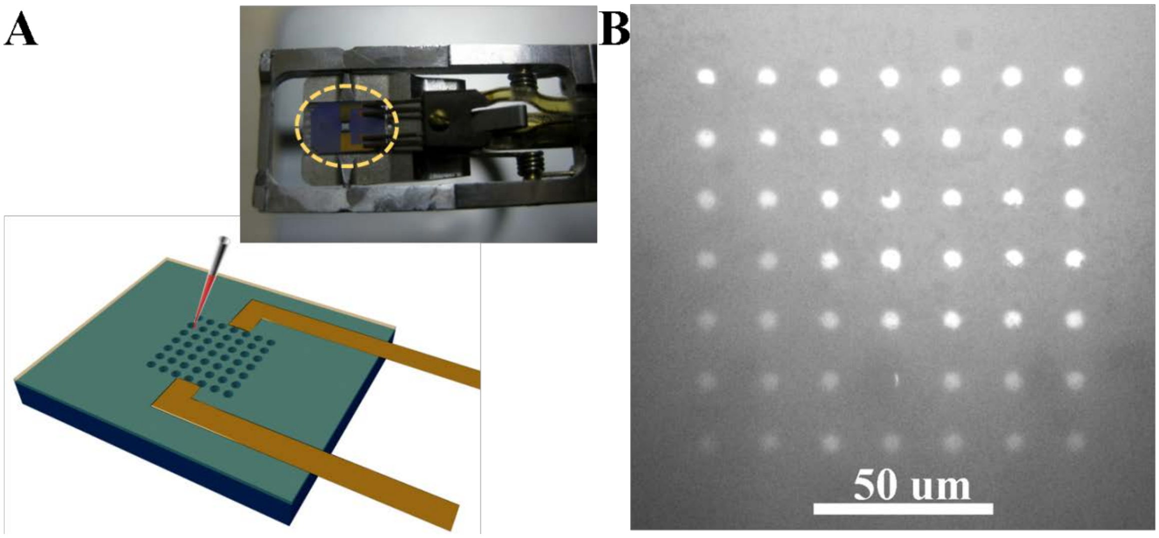

Supplementary Figure 1 | Heating E-chip. (A) The preparation of heating E-chip with precursor, and the image of mounted Protochips Aduro heating stage (inset). The yellow circle in the inset denotes the E-chip part. (B) Micrographs of the E-chip, showing the 7x7 electron-transparent holes. The Si_3N_4 heating E-chip was drop-casted with $(NH_4)_2MoS_4$/DMF solution before naturally dried in air, then mounted on the Aduro platform. Therefore, an electron-transparent thin-layer of amorphous solid precursor was formed and can be used in the following in-situ experiments.

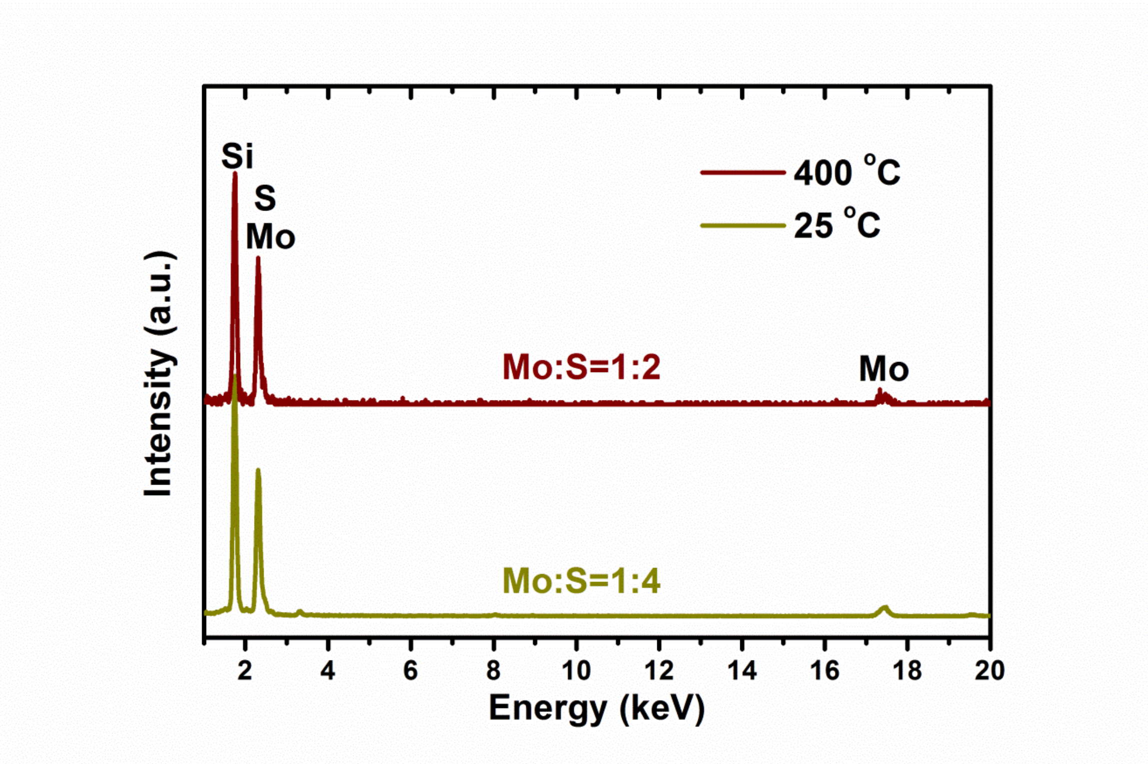

Supplementary Figure 2 | EDS analysis during the in-situ observation. The EDS spectra acquired at room temperature and 400 ^{o}C, respectively.

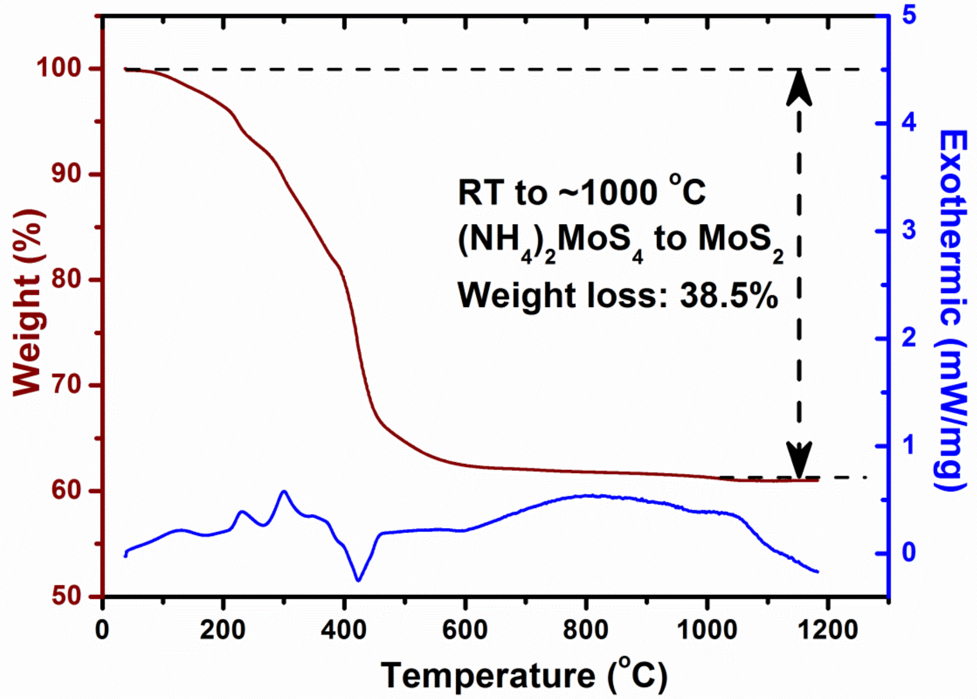

Supplementary Figure 3 | TG-DSC profiles of $(NH_4)_2MoS_4$ under flowing nitrogen. Simultaneous thermogravimetric analysis & differential scanning calorimetry (TG–DSC) analysis of $(NH_4)_2MoS_4$ precursor was conducted under flowing nitrogen before the in-situ TEM experiment. The profiles suggest a series of sub-reactions as well as continuous weight losses have happened upon heating from room temperature to ~ 1000 ^{o}C, leading to a total weight loss of 38.5% which implies a transition from $(NH_4)_2MoS_4$ to MoS_2 (The equations 1 and 2 in our article).

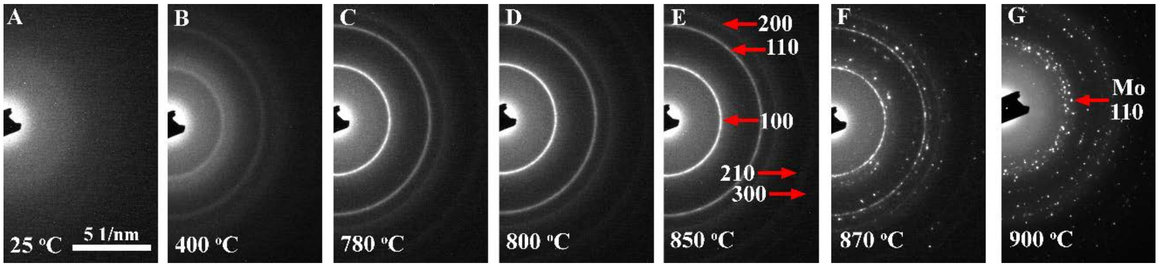

Supplementary Figure 4 | The full SAED survey, range from room temperature to 900 °C. The scale bar in (A) also applies to (B)–(G). Note that, the crystallization of MoS_2 started at 400 ^{o}C, and the SAED pattern became slightly discrete at 800 ^{o}C. A trace of Mo was identified at 870 ^{o}C, and the presence of metallic Mo became obvious at 900 ^{o}C. As can be seen in Supplementary Figure 4 B-G, the SAED pattern became sharper and brighter from 400 – 800 ^{o}C, and became more and more discrete from 800 – 900 ^{o}C. The sharper and brighter SAED pattern corresponds to better crystallinity of MoS_2, and the more and more discrete SAED pattern implies larger crystal size.

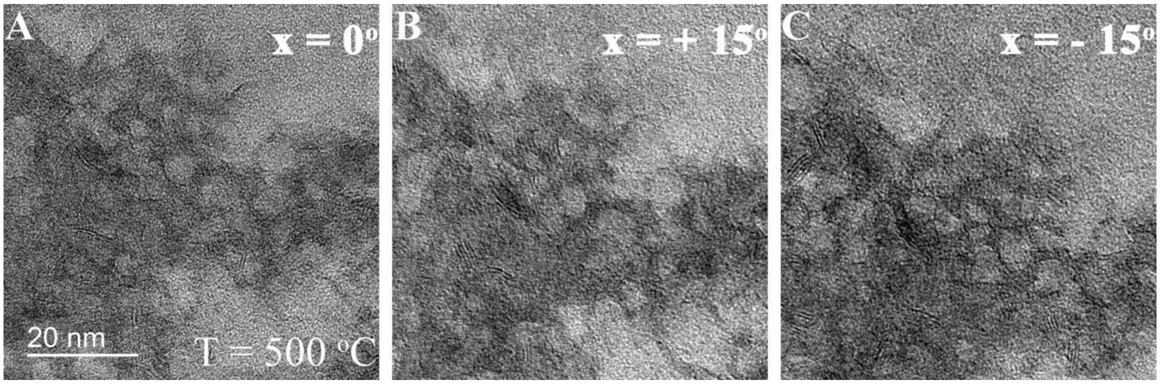

Supplementary Figure 5 | Sequential micrographs of a same place upon different tilting angles in X-axis. The images were captured at the temperature of 500 °C. The scale bar in (A) also applies to (B - C). The image contrast concerning the MoS_2 clusters significantly changes upon titling the X-axis. For example, some clusters can be found in (A) while disappear in (B) or (C), while other new clusters emerge.

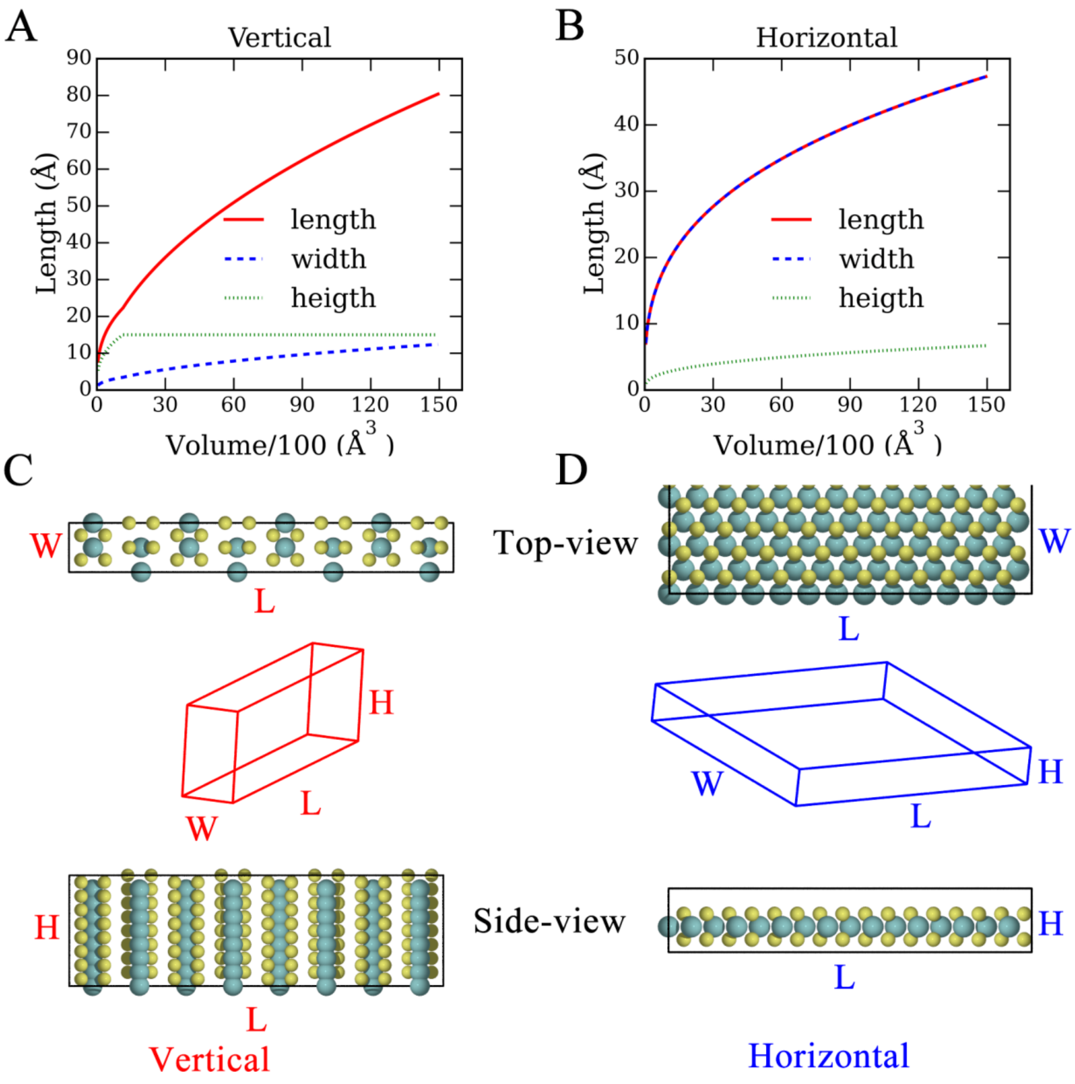

Supplementary Figure 6 | The calculation details for Figure 3G. The length, width and height of particles with minimal energy for (A) vertical and (B) horizontal orientations. Note that the length is equal to width in the case of horizontal orientations. (C) and (D) illustrate the shape and atomic arrangements for the particles with vertical and horizontal orientations, respectively.

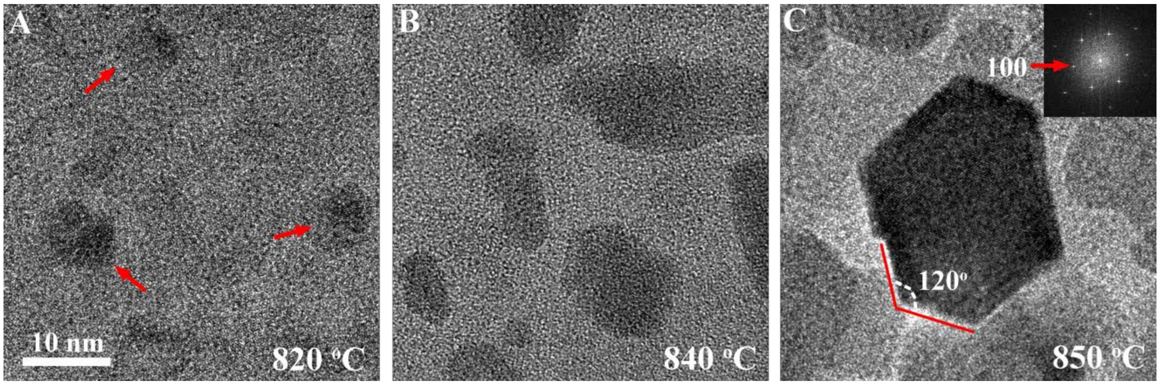

Supplementary Figure 7 | Sequential HRTEM images showing the structural transformation from irregular particles to faceted hexagonal nanoflakes. The inset in (C) is the corresponding FFT patterns. The scale bar in (A) also applies to (B - C).

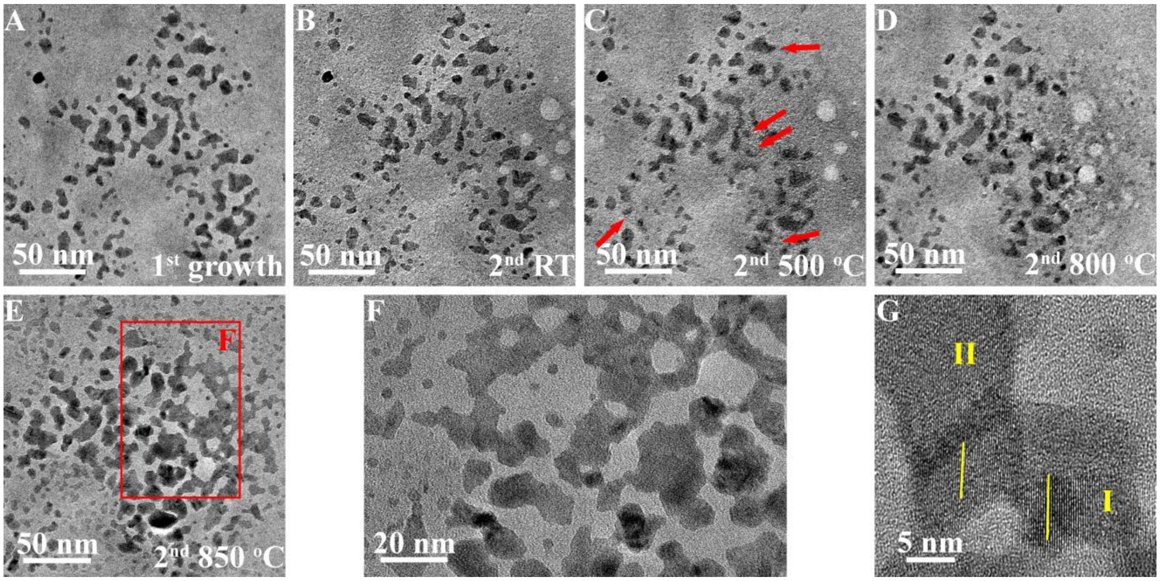

Supplementary Figure 8 | Secondary in-situ growth of MoS_2 structure. (A) TEM image for $(NH_4)_2MoS_4$ precursor annealed at 850 ^oC for 1 hour. (B) TEM image of the same position as (A) after 2nd drop of $(NH_4)_2MoS_4$/DMF solution at room temperature. (C - E) Micrographs during 2nd growth at different temperatures (500 ^oC, 800 ^oC and 850 ^oC). The red arrows in (C) indicate the vertical structures of MoS_2. (F) Enlarged image of (E) as indicated by the red rectangle. (G) High-resolution image showing the same orientation of two adjacent particles. The yellow lines denote the crystal planes for particle I and particle II, respectively.

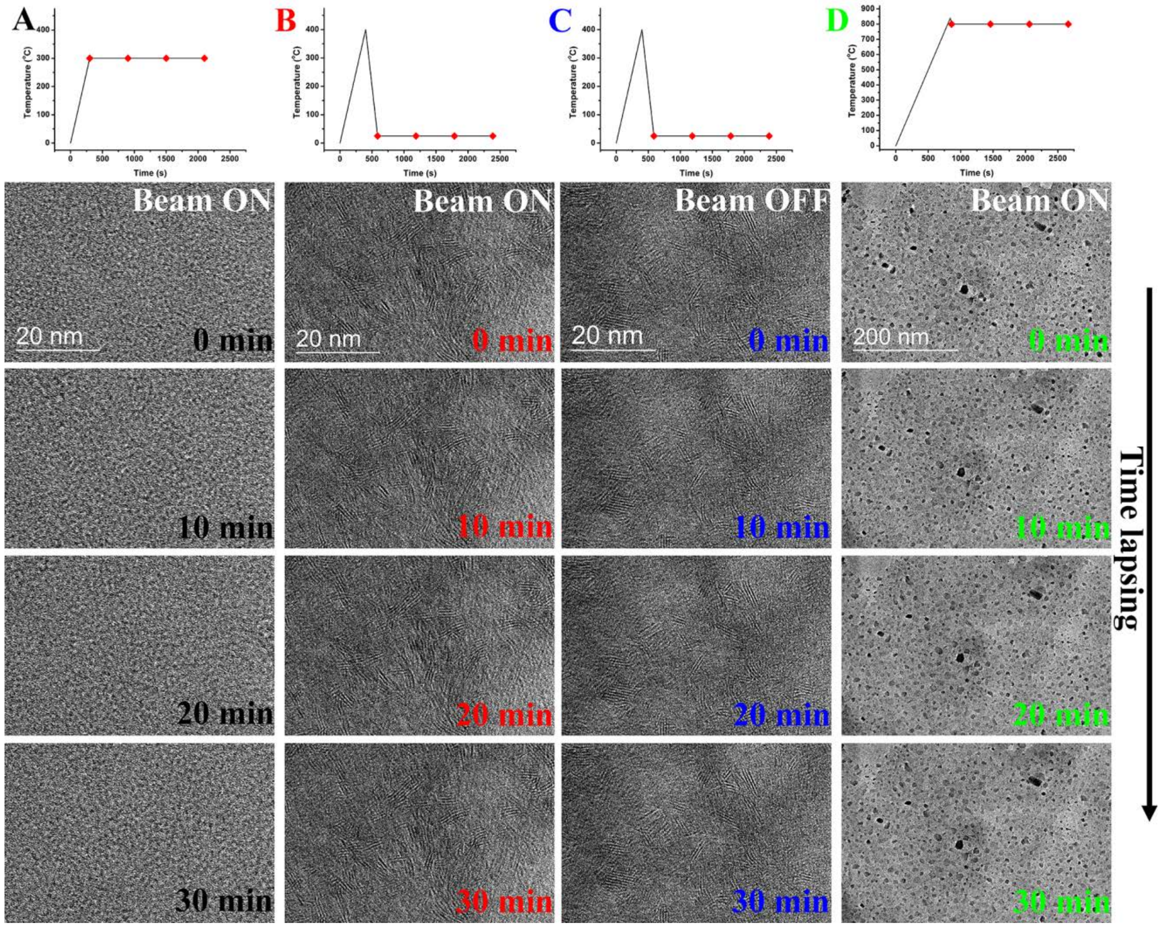

Supplementary Figure 9 | Investigation of the effect of the electron beam irradiation on the growth dynamics. The first panel of each column shows the heating program of the corresponding control experiments, and the red dots indicate the imaging moments. The scale bar in the first frame also applies to their lower frames for each column. Note there was a short period of ca. 1-2 min for sample stabilization after heating or cooling at each time before imaging, which has not been reflected by the heating programs.

Supplementary Note 1

The calculation details of surface free energies of different surfaces

The details of the present DFT calculations have been given in sub-section "Theoretical calculations" of Methods already. And the surface free energies of different surfaces are calculated following the wildly-used First-principle constraint thermodynamics approach.[1] The surface free energies of different surfaces are given by

$$\gamma = \frac{1}{2A}\left[E^{\mathrm{slab}} - N_{\mathrm{Mo}}\mu_{\mathrm{Mo}} - N_{\mathrm{S}}\mu_{\mathrm{S}}\right] \qquad (1)$$

Since the compound should fulfill thermodynamic constraint conditions $\mu_{\mathrm{Mo}} + 2\mu_{\mathrm{S}} = E^{\mathrm{bulk}}_{\mathrm{MoS_2}}$, the leading surface free energy can be written as $\gamma = \frac{1}{2A}\left[E^{\mathrm{slab}} - N_{\mathrm{Mo}}g^{\mathrm{bulk}}_{\mathrm{MoS_2}} + (2N_{\mathrm{Mo}} - N_{\mathrm{S}})\mu_{\mathrm{S}}\right]$. The range of chemical potential $\Delta\mu_{\mathrm{S}}$ is $\frac{1}{2}H_{\mathrm{f}}^{\mathrm{MoS_2}}$ (-1.322 eV) to 0 eV. This method was also used by the Norskov's[2] and Raybaud's[3] groups to investigate the stability and the corresponding phase diagram of MoS_2 in different working environments.

Supplementary Note 2

Discussions on the secondary growth of MoS_2

The secondary growth of MoS_2 was conducted on same E-chip to evaluate the possibility of growing large-area flakes in our experiment. Firstly, an E-chip with $(NH_4)_2MoS_4$ was subjected to annealing at 850 ^oC for 1 hour, leading to irregular faceted flakes (Supplementary Figure 8A) which was similar to that in Figure 4D. Then, the E-chip was taken out from the TEM and re-dropcasted with $(NH_4)_2MoS_4$ solution. After re-mounting the E-chip into TEM, the same area was located (Supplementary Figure 8B).

Consequently, the in-situ heating was re-conducted. At 500 ^oC, we can also identify MoS_2 vertical structures, appearing randomly on the membrane, as shown in Supplementary Figure 8C. Until 800 ^oC, no obvious morphological change has happened to the flakes (Supplementary Figure 8D). However, at 850 ^oC, as shown in Supplementary Figure 8E, the existing MoS_2 flakes were largely connected by newly-emerging flakes, displaying as larger flakes with faceted and quasi-hexagonal outlines (see the magnified image Supplementary Figure 8F). Furthermore, the neighboring flakes were trended to adopt same orientation, as vividly evidenced by Supplementary Figure 8G. Although we have not obtained perfect flakes throughout this experiment, the current result showed significant possibility for growing large flake with our method.

Supplementary Note 3

Investigation of the effect of the electron beam irradiation on the growth dynamics

For TEM experiments, it is essential to assess the influences from the electron beam to the experimental phenomena.[4,5] In our situation, it is also important to confirm the current results to be thermally-assisted evolution or electron-induced process. Therefore, systematical investigations on the effect of the electron beam irradiation on the growth dynamics was carefully carried out.

Firstly, the effect of the electron beam on the formation of vertical MoS_2 layers was assessed. We increased the temperature of E-chip to 300 ^oC (at a rate of 1 ^oC s^{-1}) and maintained at this temperature. The representative images were then captured every 10 minutes upon the constant irradiation of electron beam at 65 pA cm^{-2} (column A in Supplementary Figure 9). Throughout the assessment, negligible change can be identified from the precursor. No crystallized MoS_2 layer can be detected upon the electron beam irradiation for over 30 min. Subsequently, another sample was heated to 400 ^oC (at a rate of 1 ^oC s^{-1}) to form densely vertical MoS_2 clusters. Then the sample was cooled down to room temperature (at a rate of 2 ^oC s^{-1}). After that, two series of image were recorded, respectively. One position was illuminated by the electron beam (at 65 pA cm^{-2}, as column B in Supplementary Figure 9), and the other position was far away from the electron beam (except the image period of ~ 1s each time, as shown in column C of Supplementary Figure 9). Both of the image sequences showed their high stability during the observation period (30 minutes). We didn't observe noticeable sample damage or layer-by-layer growth during these two control processes. The above experiments suggest

that the thermal heating processing plays the dominant role in the dynamics of layer-by-layer vertical growth as described in our work.

Secondly, the effect of the electron beam on the particle growth process at higher temperature was also quantitatively assessed. The precursor was directly heated to 840 ^{o}C (at a rate of 1 ^{o}C s^{-1}) to form irregular nanoparticles, followed by slowly cooled down to 800 ^{o}C (at a rate of 2 ^{o}C s^{-1}). The as-formed MoS_2 nanoparticles were then illuminated by constant electron beam (65 pA cm^{-2}). Sequential images were taken every 10 minutes (column D in Supplementary Figure 9) during this process. No obvious coalescence event was observed in this 30 minutes process. We also randomly selected 20 nanoparticles in the frame and measured their size change during the assessment. As expected, the size change of the particles was within a narrow window of ± 3%, which is much less than the increase of the particle size by the heating process. These control experiments have quantitatively demonstrated that both the classical Ostwald ripening and non-classical oriented attached growth mechanisms are independent on the electron beam irradiation.

Additionally, we also carefully investigated the impact of pre-illumination of precursor in vacuum on the subsequent formation of vertical layers and nanoparticles. Before the growth of vertical structures (at 400 ^{o}C) and nanoparticles (at 840 ^{o}C), four different positions on the E-chip were separately chosen, which were illuminated by electron beam for 5 minutes at a current density of 35, 50, 65, and 80 pA cm^{-2}, respectively. Subsequently, the precursor was heated to certain temperatures (at a rate of 1 ^{o}C s^{-1}) while electron beam was turned off. After reaching the targeted temperature for a short period (ca. 2 minutes), the electron beam was turned on and the four positions were sequentially examined. In our situation, the experimental phenomena (the morphology

and density of vertical clusters, the size and shape of the horizontal particles, *etc.*) from these four positions were quite similar, which were also comparable with two previously unilluminated regions.

In summary, the above control experiments have systematically and quantitatively suggested that the as-revealed mechanisms (layer development, OA, and OR) are mostly resulted from the thermal heating process.

Supplementary References

1. Reuter, K. & Scheffler, M. Composition, structure and stability of RuO_2(110) as a function of oxygen pressure. *Phys. Rev. B* **65,** 035406 (2001).

2. Bollinger, M. V., Jacobsen, K. W. & Nørskov, J. K. Atomic and electronic structure of MoS_2 nanoparticles. *Phys. Rev. B* **67,** 085410 (2003).

3. Raybaud, P., Hafner, J., Kresse, G., Kasztelan, S. & Toulhoat, H. Ab initio study of the H_2–H_2S/MoS_2 gas–solid interface: the nature of the catalytically active sites. *J. Catal.* **189,** 129–146 (2000).

4. Simonsen, S. B. *et al.* Direct observations of oxygen-induced platinum nanoparticle ripening studied by in situ TEM. *J. Am. Chem. Soc.* **132,** 7968–7975 (2010).

5. Hansen, L. P., Johnson, E., Brorson, M. & Helveg, S. Growth mechanism for single- and multi-layer MoS_2 nanocrystals. *J. Phys. Chem. C* **118,** 22768–22773 (2014).
